# Amphetamine-induced Reverse Takotsubo Cardiomyopathy and Thrombosis: A Case Report

**DOI:** 10.5811/cpcem.40059

**Published:** 2025-11-03

**Authors:** Anthony Rabil, Omar Fakhereddine, Abdallah Rebeiz, Tharwat El Zahran

**Affiliations:** * American University of Beirut Medical Center, Department of Emergency Medicine, Faculty of Medicine, Beirut, Lebanon; † American University of Beirut Medical Center, Department of Internal Medicine, Faculty of Medicine, Beirut, Lebanon

**Keywords:** case report, amphetamine, reverse Takotsubo cardiomyopathy, thrombosis

## Abstract

**Introduction:**

Takotsubo cardiomyopathy is characterized by stress-induced left ventricular dysfunction. The reverse form accounts for < 25% of all cases. We present a case of reverse Takotsubo cardiomyopathy in a young, otherwise healthy, woman following illicit drug use

**Case Report:**

A 19-year-old female patient presented to the emergency department (ED) complaining of insomnia, left-sided chest pain, palpitations, and heightened energy levels after ingesting a significant quantity of small, rounded chocolate chips containing unidentified pills at a rave party the previous night. Her electrocardiogram revealed sinus tachycardia with ST-segment elevation in leads I and aVL. At the ED she developed respiratory distress and required oxygen supplementation. Her troponin level was 1.048 nanograms per milliliter (ng/mL) (reference range < 0.03 ng/mL), and her urine drug screen was positive for methamphetamines. Point-of-care transthoracic echocardiography showed moderately impaired left ventricular systolic function (ejection fraction approximately 35–39%) and hypokinesia of basal and mid-left ventricular segments accompanied by hyperkinesia of the apical segments, indicative of reverse Takotsubo cardiomyopathy. The patient was admitted to the cardiac care unit and showed clinical improvement after supportive treatment. However, 30 hours after discharge she presented back to the ED with epigastric pain and was found to have left renal artery thrombosis and an occlusive thrombus at the right internal iliac artery on computed tomography angiography.

**Conclusion:**

Amphetamine intoxication is associated with the development of reverse Takotsubo cardiomyopathy, along with multiple vascular thromboses.

## INTRODUCTION

Takotsubo cardiomyopathy (TTC), first described in 1980, is characterized by stress-induced left ventricular dysfunction that is reversible in most cases. It typically occurs in the setting of acute medical illness or during physical/emotional stress, hence its alternative name—broken heart syndrome.^1^ Clinical presentation resembles that of acute coronary syndrome and is marked by electrocardiogram (ECG) changes, elevated troponin, and abnormal wall motion on echocardiography, despite the absence of obstructive pericardial coronary artery disease.^2^ Several phenotypic variants of TTC have been reported, with the reverse form accounting for < 25% of all cases. Reverse TTC manifests as apical hyperkinesis alongside basal and midventricular hypokinesis or akinesis.^3^

Methamphetamine, a potent and addictive substance, is known for its modification from amphetamine through the addition of a methyl group, which enhances its lipid solubility and facilitates greater penetration through the blood-brain barrier.^4^ While the exact underlying pathophysiology is still under investigation, hypothesized mechanisms include catecholamine cardiotoxicity, coronary artery spasm, coronary microvasculature impairment, and estrogen deficiency.^2^ We present a case of reverse cardiomyopathy in a young, otherwise healthy, woman following illicit drug use.

## CASE REPORT

A previously healthy 19-year-old female, who was undergoing treatment for bronchitis with oral steroids, presented to the emergency department (ED) complaining of insomnia, left-sided chest pain, palpitations, and heightened energy levels following attendance at a rave party the previous night. She confirmed ingestion of marijuana edibles, a significant quantity of small, rounded chocolate chips containing unidentified pills, and multiple energy drinks approximately 12–14 hours prior to ED presentation. Her symptoms started three hours post-ingestion, characterized by temporary confusion, decreased concentration, incoherent speech, and severe left-sided chest and back pain radiating to her left shoulder.

**Image 1:**
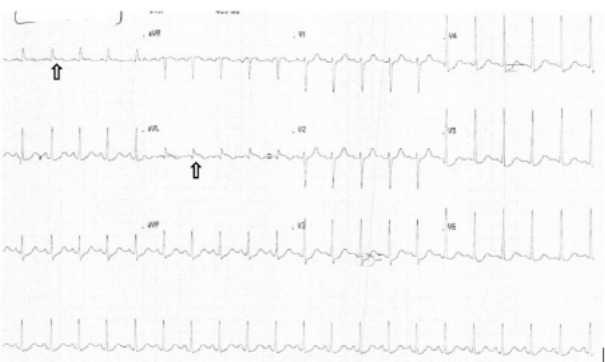
Initial electrocardiogram (ECG) showing sinus tachycardia with ST-segment elevations in leads I and aVL (arrows).

Although these symptoms resolved, she continued to experience persisting mild chest pain. Additionally, she exhibited diaphoresis, tachycardia, headache, chest tightness, and nausea but was unable to vomit. Review of systems was otherwise unremarkable. Upon presentation, she was tachycardic with a heart rate of 137 beats per minute. She appeared anxious, complaining of mild dyspnea and rapid breathing. Her pupils were dilated and reactive. The rest of her physical exam was otherwise unremarkable. Intravenous fluids were administered along with symptomatic treatment. An ECG revealed sinus tachycardia with ST-segment elevation in leads I and aVL (Image 1).

**Image 2:**
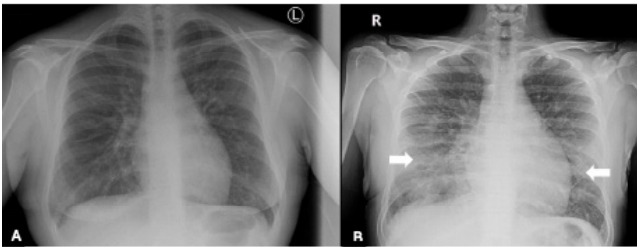
Chest radiograph on (A) initial presentation and (B) following the development of respiratory distress after intravenous hydration, demonstrating bilateral interstitial infiltrates (arrows).

A chest radiograph (CXR) displayed normal findings (Image 2A). As the patient awaited the results, she developed dyspnea, worsening tachycardia, and desaturation to 92%, while maintaining her airway. She reported increased severity of her left chest pain. Supplemental oxygen was administered, and a repeat ECG revealed no changes. A repeat CXR revealed bilateral bronchial wall thickening with patchy, interstitial-like opacities in the mid and lower lung fields (Image 2B). Intravenous (IV) hydration was withheld.

*CPC-EM Capsule*

What do we already know about this clinical entity?

*Reverse Takotsubo is a rare stress-induced cardiomyopathy variant linked to catecholamine surges, often misdiagnosed, especially in young or atypical patients.*

What makes this presentation of disease reportable?

*This rare case shows reverse Takotsubo with major arterial thromboses in a healthy 19-year-old after methamphetamine use, highlighting severe stimulant-related risks.*

What is the major learning point?

*Methamphetamine toxicity can cause reverse Takotsubo cardiomyopathy and large vessel thromboses. Clinicians must suspect cardiac issues and ensure close follow-up in young stimulant users.*

How might this improve emergency medicine practice?

*Early cardiac evaluation and awareness of delayed thrombotic risks in young stimulant users can improve diagnosis, monitoring, and safety in emergency care.*

Her troponin level was 1.048 nanograms per milliliter (ng/mL) (reference range: < 0.03 ng/mL). The urine drug screen was positive for amphetamines and tetrahydrocannabinol. The cardiology team was consulted. Point-of-care transthoracic echocardiography showed moderately impaired left ventricular systolic function with an estimated left ventricular ejection fraction (LVEF) of 35–39% (50–70%). There was hypokinesia of basal and mid-left ventricular segments, accompanied by hyperkinesia of the apical segments, indicative of reverse TTC.

The patient was admitted to the cardiac care unit, and her symptoms improved with IV diazepam as needed. Given her age, symptom improvement, and absence of concerning findings for obstructive coronary disease, cardiac catheterization was deferred. Subsequently, she was initiated on ivabradine five milligrams (mg) twice daily. Ivabradine lowers heart rate by selectively inhibiting the “funny” (*I*_f_) current—a key pacemaker current in the sinoatrial node responsible for initiating spontaneous diastolic depolarization—without affecting myocardial contractility or blood pressure.^5^

She was discharged home 48 hours later after complete resolution of her symptoms. However, 30 hours post-discharge, the patient presented back to the ED with periumbilical pain exacerbated by worsening of pre-existing constipation. Her review of system was positive for nausea. Her physical examination revealed periumbilical tenderness.

**Image 3:**
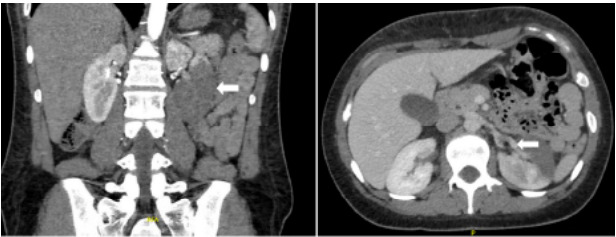
Left renal artery thrombosis with left kidney infarcts indicated by white arrow on computed tomography angiography—coronal view on the left and axial view on the right—done in the setting of epigastric pain.

**Image 4:**
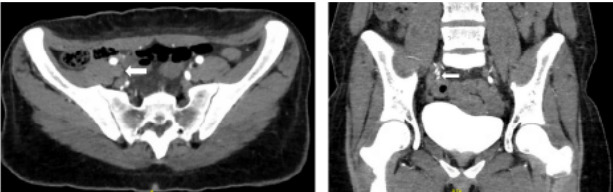
Right internal iliac artery thrombosis indicated by white arrow on computed tomography angiography—axial view on the left and coronal on the right—done in the setting of epigastric pain.

Computed tomography angiography of the abdomen demonstrated left renal artery thrombosis, with complete occlusion of the anterior segment, partial occlusion of the posterior segment, and slight extension into the main renal artery (Image 3). Additionally, secondary renal infarction was observed, characterized by increased hypodensity involving the posterior aspect of the kidney and an occlusive thrombus at the right internal iliac artery (Image 4). On blood workup, she was found to have an acute kidney injury with a creatinine increase from 0.62 mg per deciliter (dL) on discharge day to 0.95 mg/dL (0.5–1.0 mg/dL). Ramipril was withheld to avoid possible worsening of renal function.

Vascular surgery and interventional radiology teams were consulted for possible embolectomy. The family was debriefed on the risks vs benefits of intervention. Both teams agreed with the family that the best course of treatment was conservative management with therapeutic anticoagulation. The patient was started on acetylsalicylic acid and heparin drip and was admitted to the cardiac care unit for further investigation.

Point-of-care ultrasound showed normalization of left ventricular function that was still mildly dilated with a LVEF of 60%. In the investigation of thrombosis findings, a family history of deep venous thrombosis was found in the paternal uncle and aunt and from the mother’s side. (The father and mother were first-degree relatives.)

Thrombophilia workup was negative for lupus anticoagulant. Screening for factor V, factor II, and methylenetetrahydrofolate reductase (MTHFR) gene mutations using FV-II-MTHFR strip assay (reverse hybridization-sequence specific oligonucleotide probe) showed one heterozygous mutation of MTHFR (C677T) (Appendix 1). Since the patient’s creatinine level normalized, the hematology team recommended switching from heparin drip to subcutaneous enoxaparin and to follow up in clinic after discharge. She was discharged home on subcutaneous enoxaparin, bisoprolol, ivabradine, and acetylsalicylic acid.

## DISCUSSION

Takotsubo cardiomyopathy, also referred to as stress-induced cardiomyopathy, is an acute but reversible form of left ventricular dysfunction that mimics the presentation of an acute myocardial infarction. It is believed to be triggered by physical and emotional stress, although cases without identifiable triggers have also been reported.^6^ The incidence is estimated to be 15–30 cases per 100,000 per year in the United States, but this figure may be underestimated due to confusion with acute coronary syndrome and subclinical cases in patients who do not seek medical attention.^7^ Takotsubo cardiomyopathy is more common in females, particularly post-menopausal women.^6^ Several variants of TTC have been described based on the location of ventricular wall motion abnormality (akinesis or hypokinesis). The most common type is the apical variant, accounting for 81.7% of cases, followed by the midventricular type (14.6% of cases) and, finally, the basal and focal variants (2.2% and 1.5 % of cases, respectively).^6^

Reverse TTC refers to the basal type of TTC characterized by akinesis or severe hypokinesis of the base with sparing of the apex. Contrary to the apical type, reverse TTC is more commonly seen in younger rather than post-menopausal women.^7^–^9^ It has also been reported to be associated with amphetamine-type stimulants.^9^

The exact pathophysiology of TTC remains unclear. The most accepted theory is catecholamine-induced cardiotoxicity, where a surge of catecholamines and other stress hormones results in direct cardiotoxic effect and microvascular dysfunction.^2^,^10^

Methamphetamine belongs to the phenethylamine family. Its rapid onset of action and prolonged activity is related to the addition of a methyl group to amphetamine, giving it an enhanced lipophilicity.^4^ Methamphetamine acts by increasing the release of catecholamines (dopamine and norepinephrine), blocking their degradation by inhibiting the action of monoamine oxidase, and binding to various receptors of the cardiovascular system resulting in a prominent adrenergic stimulation.^11^

Reports suggest that methamphetamine causes cardiomyopathy through direct pathways such as increased production of free radicals, altered mitochondrial function and dysfunction in intracellular calcium hemostasis, and through indirect pathways by causing coronary vasospasm, hypertension, and tachycardia.^9^ Amphetamines can promote clot formation by increasing endothelial tissue factor expression, impairing natural anticoagulants and causing vascular inflammation.^12^ Moreover, although data remains limited, marijuana has been reported as a potential contributor to TTC, possibly also via catecholaminergic stimulation or autonomic dysregulation.^13^ Garakanidze et al found a correlation between MTHFR gene polymorphism and arterial thrombosis.^14^ These factors may have collectively contributed to the development of thrombosis in this patient.

Patients with TTC often present with symptoms similar to acute coronary syndrome, including angina-like chest pain, dyspnea, syncope, nausea, diaphoresis, and epigastric pain.^2^,^8^ Additionally, they may present in cardiogenic shock or dysrhythmias. On ECG, ST-segment elevations resembling those seen in ST-segment elevation myocardial infarction are common but are transient and often resolve within a few days. Other electrocardiogram findings include ST-segment depression, T-wave inversion, QT-interval prolongation, and a new bundle branch block.^8^ Patients with reverse TTC often present with ST-segment depression and QT-interval prolongation.^2^ Troponin and B-type natriuretic peptid levels are often elevated, reflecting both myocardial insult and high left ventricular pressure respectively.

Echocardiography can often distinguish between acute myocardial infarction and TTC. Cardiac catheterization with ventriculography remains the gold standard for diagnosis. The most commonly used diagnostic criteria is the revised Mayo Clinic Criteria, which include the following: transient hypokinesis, akinesis, or dyskinesis of the left ventricular midsegments with or without apical involvement with regional wall motion abnormalities extending beyond a single epicardial vascular distribution; absence of obstructive coronary disease or angiographic evidence of acute plaque rupture; new ECG abnormalities (ST-segment elevation or T-wave inversion) or modest elevation in cardiac troponin; and absence of significant stressful event including pheochromocytoma, myocarditis, intracranial bleed, or recent significant head trauma.^10^

Management of TTC is focused on supportive care and management of complications, including cardiogenic shock and arrhythmias. According to the European Society of Cardiology, patients with Takotsubo syndrome should be admitted to a monitored unit and risk stratified into high or low risk. Risk stratification is based on several criteria as shown in Appendix 2. Low-risk patients are often managed conservatively with consideration of beta-blocker therapy and angiotensin-converting enzyme inhibitors in patients with LVEF < 45%. In high-risk patients, observation is recommended in a monitored unit for at least 72 hours to assess for complications including cardiogenic shock, pulmonary edema, thrombus formation, and arrhythmias.^15^ Beta blockers can be used for management of arrhythmias, left ventricular outflow tract obstruction (with a gradient more than 40 millimeters of mercury) and when LVEF is < 45%. Angiotensin-converting enzyme inhibitors are also recommended if LVEF is < 45%. Serial imaging is helpful to reassess for improvement in regional wall motion with follow-up in 3–6 months.^15^

## CONCLUSION

Methamphetamine intoxication is associated with the development of reverse Takotsubo cardiomyopathy, along with multiple vascular thromboses. Takotsubo cardiomyopathy presents with symptoms resembling acute coronary syndrome; echocardiography can help differentiate between Takotsubo cardiomyopathy and acute myocardial infarction. Management of Takotsubo cardiomyopathy is focused on supportive care and management of complications including cardiogenic shock and arrhythmias.

## SUPPLEMENTARY MATERIAL

**Appendix 1:** Thrombophilia workup done during admission for assessment of predisposing factors of thrombosis.

Tests	Reference range	Value
Antithrombin activity	80 – 120 %	92 %
Protein C activity	80 – 130 %	111 %
Protein S activity	60 – 130 %	94 %
DRVVT (patient / normal)	--	38.6/38.8
Ratio	< 1.2	0.99
ACA IgM	< 7.0 MPLU/mL	< 7.0
ACA IgG	< 10 GPLU/mL	< 10.0
Fibrinogen	1.70 – 4.00 g/L	4.59
PT patient	10.0–13.0 seconds	13.3
INR	0.9 – 1.2	1.2
PTT (patient / control)	27.0 – 39.0	43.0/27.9

*PTT*, partial thromboplastin time; *DRVVT*, diluted Russell viper venom time; *ACA*, anti-cardiolipin antibodies; *IgM*, immunoglobulin M; *IgG*, immunoglobulin G; *PT*, prothrombin time; *INR*, international normalized ratio; *mmHg*, millimeter of mercury.

**Appendix 2:** Risk stratification in Takotsubo cardiomyopathy. Adopted from the European Society of Cardiology Guidelines.

Major Risk Factor	Value/Presence	Minor Risk Factor	Value/Presence
Age	≥ 75 years	Age	< 70–75 years
Blood pressure	< 110 mmHg	Physical stressor	+
LV function	< 35%	LV function	35–45%
Pulmonary edema	+	Biventricular involvement	+
Arrhythmias/Syncope	+	Concomitant obstructive CAD	+
LVOTO	≥ 40 mmHg	NT- proBNP	≥ 2000 pg/ml
Moderate to Severe Mitral Regurgitation	+	BNP	≥ 600 pg/ml
Apical Thrombus	+	QTC ≥ 500 ms	+
New/Contained VSD	+	Pathological Q-waves	+
		Persistent ST-segment elevation	+
High risk: at least 1 major + 2 minor criteria			

*LV*, left ventricle; *LVOTO*, left ventricular outflow tract obstruction; *VSD*, ventricular septal defect; *CAD*, coronary artery disease; *NT-proBNP*, N-terminal pro B-type natriuretic peptide; *BNP*, B-type natriuretic peptide; *QTC*, corrected QT-interval in milliseconds.
